# A neuropsychological study of chromatin disorders

**DOI:** 10.1192/bjo.2021.119

**Published:** 2021-06-18

**Authors:** Benjamin Geers, Siddhartha Banka, Daniel Weisburg

**Affiliations:** 1 University of Manchester; 2 Manchester University NHS Foundation Trust

## Abstract

**Aims:**

Analyse neuropsychological assessment data collected from a chromatin disorder clinic to determine the neuropsychological profile associated with chromatin disorders. Assess for differences in neuropsychological profile by diagnostic group and gender. Hypothesis: A systematic neuropsychological review of chromatin disorders will reveal previously unknown patterns.

**Background:**

Chromatin disorders (CD) are a group of genetic conditions that result in developmental delay and intellectual disability. Thus far the neuropsychological profile of CDs has been poorly studied.

**Method:**

Cognitive functioning, adaptive behaviour, psychosocial difficulties and perceived impact on the family were systematically assessed in a cohort of 42 patients with CDs from November 2016 to February 2019. Cognitive functioning was assessed via Full-Scale Intelligence Quotient (FSIQ), adaptive behaviour was assessed via Vineland's Adaptive Behaviour Scores (VABS), anxiety and depression was assessed via the Revised Children's Anxiety and Depression Scale (RCADS) and communication skills were assessed via the Social Responsiveness Scale-2 (SRS-2). Family Impact Scale was used to assess for the perceived impact on the family. Mean scores for each neuropsychological domain were calculated firstly sorting patients by diagnosis, and then by gender. Unpaired t-tests were run to asses for statistically significant differences in mean scores by diagnosis and gender. Spearman's correlation was used to determine and potential correlations between FSIQ, VABS, RCADS and SRS-2 scores and Family Impact Score.

**Result:**

Patients with CDs were found generally to have mild intellectual disability (mean FSIQ = 64.57) and markedly deficient adaptive behaviour functioning (mean VABS = 50.19). Patients had a mean SRS-2 score of 70, indicative of high rates of autism spectrum disorder associated symptoms. RCADS and SRS-2 scores were negatively correlated with Family Impact Score with statistical significance (–0.562 and –0.429 Correlation coefficient for RCADS and SRS-2 respectively). Females had statistically significant average higher RCADS scores than males. CHARGE Syndrome was frequently an outlier having a mean higher FSIQ score, lower adaptive functioning and lower psychosocial impairment; however, these differences were not statistically significant.

**Conclusion:**

Adaptive behaviour functioning of patients with CDs is lower than expected for their FSIQ. Females with chromatin disorders have higher levels of anxiety and depression than males however the reasons for this are unknown. The psychosocial challenges and family's impact should be considered in the clinical management of CDs. Further research with a larger data set is needed to identify the neuropsychological profiles of different CDs and to confirm whether the observed differences in CHARGE Syndrome are significant.

